# The relationship between exercise-related expectancies and exercise behaviour in adolescent athletes

**DOI:** 10.1080/21642850.2024.2356777

**Published:** 2024-05-26

**Authors:** Katharina Borgolte, Martin Pinquart

**Affiliations:** Developmental Psychology, Philipps-University Marburg, Marburg, Germany

**Keywords:** Exercise-related outcome expectancies, adolescents, sports behaviour, youth health, physical activity

## Abstract

**Background:**

In recent years, a decline in sports behaviour among adolescents was observed, even though it is generally known that sports contribute to healthy development. According to the social cognitive theory of Bandura, outcome expectancies play an important role in the practice of health behaviour.

**Methods:**

This study analysed the relationship between exercise-related outcome expectancies (EOEs) and exercise behaviour among adolescent athletes, and the differences of EOEs according to age, gender and type of sport played. In an online survey *N* = 223 (female = 140, male = 83) athletes between the age of 10–19 (*M_age_* = 14.27, *SD *= 3.21) completed the Exercise-related Outcome Expectancies Questionnaire for Adolescents (EOEQ-A), as well as questions about their sociodemographic background and training behaviour.

**Results:**

A small positive correlation of psychological EOEs with the overall amount of training was discovered while expectancies about negative consequences of sports were associated with lower training efforts. Athletes between 14 and 16 years reported significantly stronger negative EOEs than younger or older participants. Regarding gender, no significant differences were found. Furthermore, a positive correlation between social EOEs and participation in team sports, as well as training in a group was found.

**Conclusion:**

These results could help with adapting exercise-related interventions so that the positive expectancies of the athletes can be fulfilled and exercise behaviour among adolescents can be promoted. Future studies should investigate the relationship between changes in expectancies and changes in exercise behaviour.

## Exercise-related outcome expectancies and sport behaviour

In Germany, less than 30% of the adolescents reach the 60 minutes of daily physical activities recommended by the World Health Organization (Finger et al., 2018). Nevertheless, around 70% of adolescents claim to exercise in their leisure time, with boys significantly more active than girls (Finger et al., 2018). However, with increasing age, less and less time is invested in sports and exercise (Allen & Laborde, 2014; Manz et al., 2016). Therefore, it is very important to promote exercise, especially among adolescents. According to social cognitive theory (Bandura, 1986), one way to influence exercise and other health behaviours is through outcome expectancies (OE). OE are understood as the expected consequences of a certain behaviour (Williams et al., 2005). Bandura (2004) distinguishes between self-evaluative (emotional satisfaction and self-esteem), physical (body-related experiences as well as material losses and/or benefits) and social (social contact and recognition) outcome expectations. All three types of OEs include positive and negative expectancies (Bandura, 2004).

The impact of exercise-related outcome expectancies (EOEs) on sports behaviour is not yet fully understood. Some research showed that adults’ EOE predict the intention to work out, even after controlling for other predictors, such as self-efficacy or social cognitions. Moreover, if positive EOEs are not met, sports behaviour decreases compared to conditions when positive expectancies were not violated. In the case of negative EOEs, only a small effect of expectancy violations has been found for adults (Williams et al., 2005).

In the school context, the link between EOEs and a child’s practice has already been confirmed. The more favourable the expectancy and its value for the students the greater their performance, effort and persistence (Read, 2017). Furthermore, a review showed a positive association between exercise and related OE in adolescents, while the results were inconsistent for children (Sterdt et al., 2014). A study on Iranian female adolescents indicated that EOEs are correlated with exercise, not only in western countries (Taymoori et al., 2010). While adolescents’ participation in within-class sports activities has been positively correlated to the expected level of success and enjoyment, these findings could not be replicated for after-class physical activities (Read, 2017).

Previous studies have also looked at exercise-related OE among adolescent athletes. Those OE ensured that adolescent athletes work more persistently and develop a never-give-up attitudes but also led to self-doubts (Stornæs et al., 2023) as well as burnouts (Sorkkila et al., 2017).

EOEs of children and adolescents arise, among others, through a combination of support from parents, own choices, and own exercise-related experiences (Read, 2017). Since correlates of EOEs in adolescent athletes are not yet totally understood, despite probably playing an important role in the execution of sports behaviour, the aim of this study is to analyse correlations between these expectancies and the exercise behaviour among adolescents. Thereby, the sports behaviour of adolescent athletes should be better understood thus allowing for the future promotion of a continued maintenance and/or increase in sports activity.

### Gender differences

Research showed that boys and adult men work out more, on average, than girls and women across all age groups in Germany (Finger et al., 2018). Boys and adolescents seem to enjoy exercise more compared to their female peers and see themselves as more competent and able to overcome potential barriers (Best et al., 2017). Also, male athletes report more perceived competence, self-esteem and physical self-worth than girls (Inchley et al., 2011). Therefore, it is not surprising that female adolescents are driven more by extrinsic motivation for sports compared to their male peers (Yazıcı, 2016). Furthermore, an older study found that girls choose to participate in sports more often because of social reasons while boys indicate reasons such as mastery and competitions (Eccles & Harold, 1991).

### Age differences

Adolescence is defined as the time between childhood and adulthood. It covers the time span between 11 and 18–20 years, and it is associated, among others, with large changes in individual behaviours (Er, 2020). Especially the changes between adolescences and adulthood are important because they can influence behaviour till late adulthood (Corder et al., 2019). It is not surprising that also sport behaviour changes. During childhood up to the age of 12 years, adolescents try a variety of sports that are particularly fun for them. Around about until the age of 16 years, adolescents tend to focus on two to three sport types. Afterwards, competition tends to become more important, in mostly one sport type the individual chooses to focus on (Bailey et al., 2013).

Even though exercise is essential for health and well-being (Becker et al., 2006; Read, 2017), the average time invested in sports decreases during adolescence, and many adolescent athletes give up working out during that time span (Allen & Laborde, 2014; Manz et al., 2016). The lower the satisfaction of needs for relatedness and autonomy, the more likely an athlete drops out of sports (García Calvo et al., 2010). The extent to which exercise-related expectancies change across adolescence is not yet fully understood. Studies showed that the predictors of sports behaviour change with age (Sterdt et al., 2014) as well as the reasons for participating in sports (Brown et al., 2017). Moreover, some studies report age differences, while others do not (Er, 2020).

### Differences between group sports and individual sports

Exercise can be executed alone or in a team. Athletes participate in different sport types for different reasons (Molanorouzi et al., 2015). Individual sports are mostly played out of goal oriented reasons like fun (Paradis et al., 2014). Team sports, on the other hand, are more likely to prevent the athletes from anxiety and depression and therefore improve health more from the social interactions that come along with such participation (Eime et al., 2013; Inchley et al., 2011; Sabiston et al., 2016). Hence, the execution and/or the training context (alone or as a group) may influence EOEs, especially the social ones.

Molanorouzi et al. (2015) categorized exercise into team, individual, racquet, martial arts, and exercise sports. Adult athletes, who mainly participated in team sports, differed from all the other athletes in their motive for affiliation. Athletes who participated in individual sports, such as racing, had a higher motive for enjoyment than the rest of their sample. Moreover, group athletes had, in general, greater expectancies than individual athletes. Also, those who participated in both individual and team sports together indicated greater expectancies than those who only did individual sports (Yazıcı, 2016).

### Hypotheses

Based on reviewed theoretical considerations and empirical results, this study addressed the following research questions and derived hypotheses: Do EOEs vary by gender, age, sport type (individual versus team sports), weekly amount of training and training setting (alone or as a group)? The following hypotheses were tested: Adolescent athletes who train more have greater positive EOEs than those who train less (Hypothesis 1). Male adolescent athletes have greater positive EOEs than female peers (Hypothesis 2). Because exercise activities tend to decline across adolescence, Hypothesis 3 states that older adolescent athletes have less positive EOEs. Adolescent athletes with greater social-related EOEs are more likely to play team sports (Hypothesis 4). Finally, adolescent athletes who have greater social EOEs train mainly in teams rather than alone (Hypothesis 5).

## Methods

An online survey was used to investigate the cross-sectional relationship between EOE and exercise in adolescent athletes considering age, gender and training behaviour.

### Participants

Due to contact restrictions related to the COVID-19 pandemic, the study was conducted as an online survey. Adolescents between 10 and 19 years were invited to participate. Recruitment took place via sports clubs, which then forwarded the questionnaire to their members or the parents of the members, respectively. Adolescents were recruited from June 2021 to October 2021. In the case of the minor participants, parents first had to confirm by mouse click that they agreed with their children's participation. They then received the link for the survey, which they could forward to their children. Adolescents of full age were allowed to fill in the survey directly.

To participate, adolescents must have been between the ages of 10 and 19, have sufficient German skills, as well as their parent’s consent (in the case of minor participants). Participants attended voluntarily and with the opportunity to win a 25 Euro voucher or get a 35% rebate code for a sporting goods company. The study was approved by the ethical board of the psychology department of the Philipps-University Marburg (Reference number: 2021–29k). The anonymity and integrity of participants are protected in concordance with a data protection concept according to the European General Data Protection Regulation. All participants and their legal guardians were informed about the purpose and methods of the research prior to the start of the study and gave their consent to participate. A total of 236 adolescent athletes participated. Of these, 223 completed the entire questionnaire (mean age = 14.27, *SD *= 3.21; female = 140 male = 83, divers = 0, Table 1). Given the recruitment via sports clubs, all those adolescents claimed to participate in sports during their leisure time.

**Table 1.** Sample characteristics.

Variable	*n*	%
*Age*		
10	45	20.18
11	28	12.56
12	12	5.38
13	9	4.04
14	11	4.93
15	21	9.42
16	20	8.97
17	31	13.90
18	28	12.56
19	18	8.07
*Gender*		
Female	140	62.78
Male	83	37.22
Diverse	0	0
*Athlete status*		
Athlete	223	100
Non-athletes	0	0
*Type of sport*		
Team sports	132	59.19
Individual sports	91	40.81
*Type of training*		
Training in a group	169	75.78
Training alone	54	24.22

Note: *N *= 223.

### Measures

Participants were asked about their sociodemographic characteristics, such as age, gender, school type, and about their sports and training behaviour. This included whether they participated in sports outside of school and if so, which ones. It was possible to choose between 23 different sports. They indicated how often they usually train, how long a training session lasts and whether they mainly train alone or in a group, as well as whether they mainly do team or individual sports.

The Exercise-related Outcome Expectancies Questionnaire for Adolescents (EOEQ-A) by Borgolte and Pinquart (in press) has been used to capture exercise-related outcome expectancies. This questionnaire contains five scales which are almost identical to those proposed by Bandura (2004): 1. *Psychological* (emotional satisfaction and promoting sense of self-worth; e.g. having fun), 2. *Negative* (heterogeneous negative effects, e.g. getting hurt or feeling pain; e.g. having pain), 3. *Physical* (positive effects on fitness and physical health; e.g. losing weight), 4. *Social exercise-related outcome expectancies* (positive contact with peers; e.g. strengthen friendships) and 5. *Competition-related outcome expectancies* (e.g. winning contest). By inverting the second scale and adding up the first four scales, an overall value for exercise-related expectancies (expectancies in general) can be calculated. Greater values represent stronger positive expectancies. The competition scale, which can only be completed by those who participate in competitions, was not used in the present study. All together the remaining questionnaire contains 31 items (12 on the psychological scale, 11 on the negative scale, 5 items on the physical scale and 3 on the social scale).

In the present study, good internal consistencies were achieved for *psychological* (0.90), *negative* (0.83)*, physical* (.82), and *social* (0.82) *outcome expectancies.*

### Statistical analyses

The first hypothesis was tested by calculating correlations and the second and third hypotheses were tested with a Multivariate Analysis of Variance (MANOVA) and follow-up Analyses of Variance (ANOVA) with EOEs as the dependent variable and age and gender as the independent variables. Age was grouped into three groups according to Brown et al. (2017); the first group comprised the age from 10 years to 13 years, the second group from 14 years to 16 years and the last group covered all participants 17 years and older.

Hypotheses 4 and 5 were analysed by calculating an 2 × 2 ANOVA, with social EOEs as the dependent variable and training (alone versus as a group) as well as sport type (individual vs. team sports) as independent variables. The data were analysed with RStudio 3.6.3 (R Core Team, 2020).

## Results

Training amount (training days per week x duration of training session) did neither significantly correlate with the overall EOEs (all four expectancies domains summed up) (*r *= −0.01, *p *= 0.86), nor with physical (*r *= 0.11, *p *= 0.12), and social expectancies (*r *= −0.05, *p *= 0.48). In contrast, the correlations of psychological expectancies with training amount (*r *= 0.25, *p* < 0.001), number of training days (*r *= 0.24, *p* < 0.001), and average duration of training session (*r *= 0.15, *p *= 0.03) were significant even though both effect sizes were small. Also, a small but negative association of negative expectancies with training amount (*r* = −0.27, *p* < 0.001), number of training days (*r* = −0.26, *p* < 0.001), and average duration of training session (*r *= −0.14, *p *= 0.03) were found.

The MANOVA revealed a significant result (*F(4) = 2.48, p = 0.012*). The follow-up ANOVAs showed that there were significant age differences (*F*(1) = 14.93, *p *< .001) in negative EOEs. The adolescent athletes between 14 and 16 years reported stronger negative EOEs (*M *= 3.86, *SD *= 0.67) than older (*M *= 3.37, *SD *= 0.78) as well as younger athletes (*M *= 3.83, *SD *= 0.77). All effect sizes ranged from Cohen’s *f* = 0.26 to *f* = 0.023. Interestingly, girls reported weaker negative EOEs than their male peers did, even though the results were not significant. The results of the follow-up ANOVAs are shown in Table 2.

**Table 2.** Association of EOEs with age and gender (ANOVAs).

	Depended variable	Df	*F*	Sig.	Cohen´s f
Age	Psychological EOEs	1	1.002	.32	.067
	Negative EOEs	1	14.93	<.001	.260
	Physical EOEs	1	1.822	.16	.135
	Social EOEs	1	.684	.41	.056
	Overall EOEs	1	1.015	.32	.068
Gender	Psychological EOEs	1	.16	.69	.027
	Negative EOEs	1	1.933	.17	.094
	Physical EOEs	1	.273	.60	.035
	Social EOEs	1	.113	.74	.023
	Overall EOEs	1	1.727	.19	.088

Note: Df = degree of freedom, *F* = test for statistical significance.

**Figure 1.**
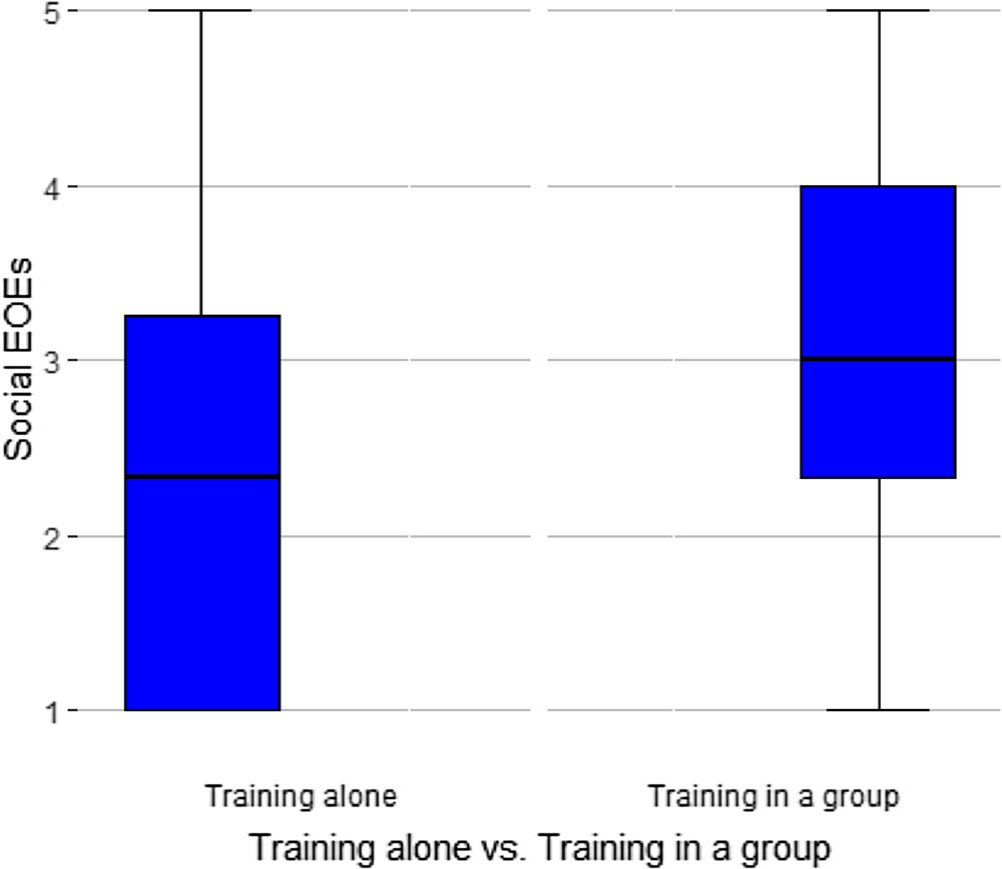
Boxplot of social EOEs related to training alone versus in a group.

**Figure 2.**
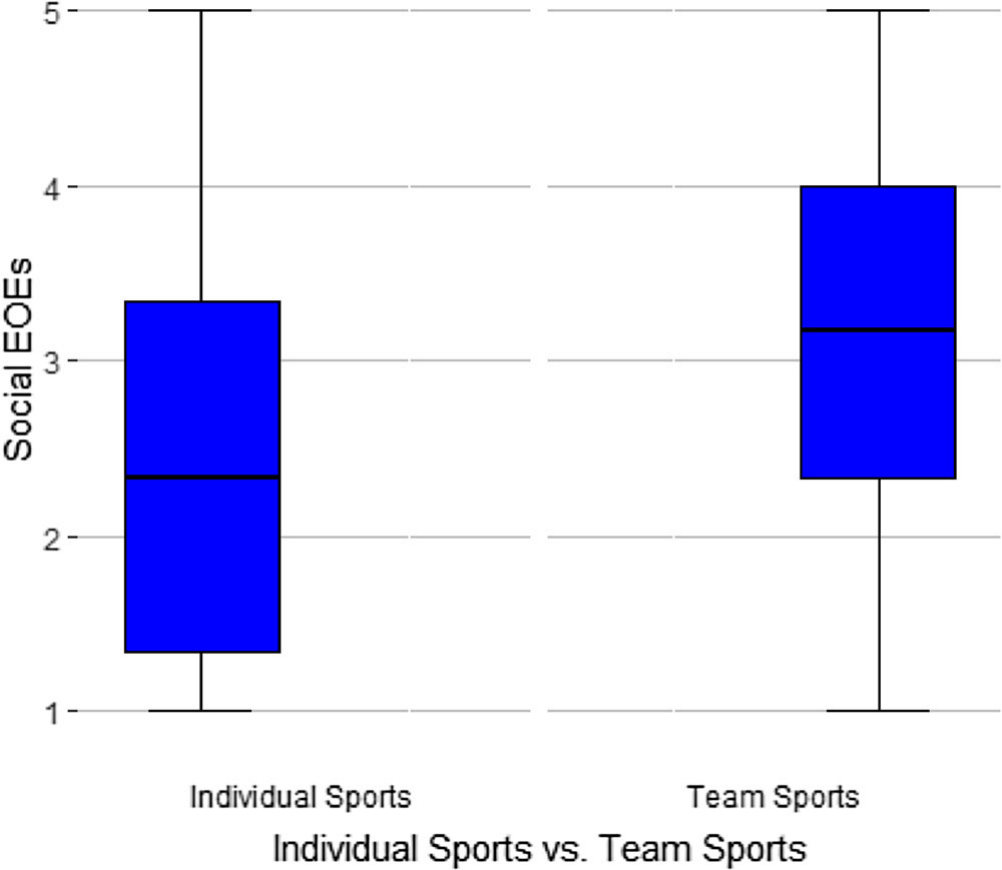
Boxplot of social EOEs related to individual versus team sports.

For testing whether adolescent athletes with greater social EOEs are more likely to play team sports and mainly train in teams rather than alone, two univariate ANOVAs were conducted with social EOEs as dependent variable and the participation in team sports and training as a team as independent variables. Both participation in team sports (*F*(1,221) = 15.41, *p* < .001) and training in a group (*F*(1,221) = 17.8, *p* < .001) had a significant statistical association with social EOEs. Those adolescent athletes who train mostly alone (*M *= 2.30, *SD *= 1.21) and those who do individual sports (*M* = 2.52, *SD *= 1.21) had lower social EOEs than peers who work out in groups (*M* = 3.08, *SD* = 1.15) and do exclusively team sports (*M *= 3.15, *SD *= 1.14), as shown in Figures 1 and 2.

## Discussion

The purpose of the present study was to assess the relationship between EOEs and exercise behaviour. For this purpose, EOEs, training variables and sociodemographic variables were recorded in an online survey among adolescent athletes. We expected those adolescent athletes who train more to have higher positive EOEs, and we found a positive correlation between the psychological EOEs and sport activity, and a small negative correlation of negative EOEs with the amount of training one does. This indicates that adolescent athletes’ psychological EOEs, such as expecting to *clear their head during sports*, are the most important EOEs to work out. On the other hand, the higher their negative EOEs, the less adolescent athletes do sports. The lack of significant associations of other EOEs with doing sports may be explained by only assessing athletes which probably restricted the variance of the EOEs.

The observed lack of an association between physical EOEs and sports behaviour could also be explained by results from an older study, which showed that besides sports, male adolescents may also resort to dieting or steroids to change their bodies (Drewnowski et al., 1995). The use of other behaviours that can meet the same expectancies as exercise could influence the association of physical EOE with sports behaviour. Moreover, for girls in particular muscles built up through exercise often do not match society's ideals of beauty which could reduce associations between physical exercise and expected effects on the body (Steinfeldt et al., 2013). Furthermore, the training intensity was not recorded here, which could, however, be a decisive point.

In terms of social EOEs, peers become more important at the beginning of adolescents. For older adolescents’ romantic relations gain importance (Eime et al., 2019). Expectancies about contact with peers may not be very relevant for the amount of exercising in those adolescent athletes who also have many social contacts outside the sports context or who conduct mainly individual sports (Reichter & Weiss, 2018). The observed lack of a significant correlation of social EOEs with sport activities may also be based on COVID-19-related social restrictions at the time of data collection which limited informal contact of the athletes.

Furthermore, gender and age were expected to be associated with EOEs. We found no significant differences regarding the EOEs. This might contradict previous studies showing that in Germany boys tend to do more sport and enjoy it more than their female peers do (Best et al., 2017; Finger et al., 2018). In both cited studies, male adolescents have been more active than their female peers whereas in the present study, both the boys and the girls were active in sports almost equally. This could explain the differences between previous studies and the present study. In addition, male adolescents seem to be realistic when expecting more negative side effects of sports than their female peers. More concretely, male athletes in general (Henke et al., 2014) as well as male adolescents in particular tend to experience more accidents during sports because of their riskier behaviour (Woller & Ellsäßer, 2014). Due to the more frequent negative experiences, the negative EOEs would increase too.

According to the age of adolescent athletes, a nonlinear association with negative EOEs was observed. The peak of negative EOEs in middle adolescence parallels age differences in risky behaviours due to a temporal imbalance between reward sensitivity and impulse control (Steinberg, 2010). Most negative physical side effects of sports, such as injuries due to attempts of impressing their peers, may be expected in middle adolescence. Late adolescents are able to assess their own exercise performance more realistically (Eime et al., 2019) which could reduce expected and factual negative consequences of doing sports, such as demanding too much from oneself and feeling pain. The drop in negative EOEs in 17-year-old and older adolescent athletes may also result from a selectivity effect indicating that only those athletes stayed in sports and continue to do so who have less negative EOEs.

A statistically significant relationship of the social EOEs with participation in team sports as well as training in a group was assumed and also found. It is hardly surprising that adolescent’s expectancies regarding sports influence the type of sport that is practiced and that the type of sports affects their future expectancies. It makes sense that those who like to spend time with friends playing sports are also more likely to play together in a team and/or train together with them. These results are also consistent with previous studies in which team athletes had higher expectancies (Yazıcı, 2016) and cared more about affiliation (Molanorouzi et al., 2015). As peer relationships become more important from early to mid-adolescence, it would be interesting to find out in longitudinal studies whether the correlation between exercise behaviour and social EOEs also changes with age. These findings are especially important for planning interventions to keep adolescent athletes on working out.

### Limitations

One of the limitations is the limited sample size, and the limited number of types of exercise studied and the accompanying difficulty regarding the generalization of the results. Further, this study only covered athletes. Therefore, it has not been possible to make a statement about the relationship between the expectancies and exercise behaviour in mixed samples of athletes and non-athletes. The variance of the exercise participation as well as EOEs could be restricted by the fact that only athletes participated in the study. This variance restriction may have reduced the size of correlations between EOEs and sport activities. Another limitation is the lack of longitudinal data that could have examined the statistical effects of EOEs on change in sport activity as well as of activity on change in EOEs.

A further limitation is that daily variations in training duration and intensity were not recorded. Furthermore, no detailed data on the training sessions was collected; only how long the training sessions lasted on average and how often they normally trained per week had been assessed.

Furthermore, this study has been conducted during the COVID-19 restrictions in Germany. Even though sporting activities in clubs were possible again at the time of our data collection, many precautionary measures were in place that could also have influenced the sporting lives of adolescent athletes as for example the social distancing recommendations. The restrictions could also have led to a reduction in sports participation or a switch to alternative forms of physical activity due to uncertainties regarding whether and when COVID-19-related restrictions of some sports activities will be lifted.

### Theoretical conclusion

This study demonstrates that there is a connection between psychological and negative EOEs with the sport behaviour of adolescent athletes. This shows that especially the psychological EOEs are important for sports participation among adolescent athletes and that the negative EOEs probably have the greatest influence on dropping out of exercise or for a lower level of sports activity. For physical and social EOEs, on the other hand, no statistically significant correlation with exercise could be found. Bandura (1986) generally assumes a connection between expectancies and health behaviour. However, a differentiated view must be taken here, as this study shows that, at least in the case of adolescent’s exercise behaviour, not all outcome expectancies may have a significant influence on sporting behaviour.

We conclude that male adolescent athletes tend to expect a similar amount of negative consequences of exercise as their female peers, probably reflecting their higher risk for accidents and physical overload to impress others. The negative EOEs are greatest for the middle-aged adolescent athletes which could be because of the temporal imbalance between reward sensitivity and impulse control and the related peak of risky physical activities in that age group (Steinberg, 2010).

Furthermore, similar to previous studies we conclude that the social EOEs associate with the training amount as well as the way the athletes train (alone or in groups) and which sport they participate in (team or individual sport).

### Practical conclusion

Understanding the expectancies adolescent athletes have towards their exercise behaviour is essential to develop interventions to reduce the high rate of dropouts in sport activities among adolescent athletes. When planning interventions, special attention should be paid to psychological EOEs, which have the greatest correlation with adolescent athletes’ exercise behaviour. Age should also be considered, as adolescent athletes between 14 and 16 years of age have more negative EOEs than younger and older adolescent athletes. Moreover, those adolescent athletes for which peer relationships are particularly important should be attracted with the help of team sports. For those for whom other EOEs are important, it is necessary to look individually which sport can best meet their EOEs. By adapting the choice of sport, the number of exercise dropouts could be reduced, and more adolescent athletes could be encouraged to participate more in sports.

### Conclusion for future research

Various kinds of studies should be conducted in the future. For example, the inverse correlation between negative EOEs and the sport behaviour indicates that longitudinal studies are needed for testing the predictive effect of negative EOEs and of the fulfilment of these EOEs on the reduction and abandonment of sport activities. Whether the fixed duration of some training sessions of sports clubs decreases the size of correlations of expectancies with duration could also be examined in more detail in future studies.

While the present study only assessed average age differences in EOEs, future longitudinal studies should test whether there are subgroups with different trajectories and which factors contribute to these trajectories, such as changes in sport activities or in self-control skills needed to find optimal levels of challenges. Finally, EOEs of non-athletes should be inspected to identify some barriers that must be overcome for promoting physical activities of these individuals.

## Supplementary Material

Supplemental Material

## Data Availability

The data that support the findings of this study are available from the corresponding author [borgolte@staff.uni-marburg.de], upon reasonable request.
