# Supplementary material for: The relationship between exercise-related expectancies and exercise behaviour in adolescent athletes
Source: Health Psychol Behav Med. 2024 May 26;12(1):2356777. doi: 10.1080/21642850.2024.2356777 (PMC11132558; doi:10.1080/21642850.2024.2356777)
Supplement: Supplemental Material [file RHPB_A_2356777_SM1577.docx]

**Supplementary Material**

**Table1**

*Correlations Between Study Variables*

|  | Training-  Days | Training-  Duration | Age | Social EOEs | Physical EOEs | Negative EOEs |
| --- | --- | --- | --- | --- | --- | --- |
| Psychological EOEs | 0.08 | 0.01 | 0.06 | 0.51** | 0.75** | 0.55** |
| Negative  EOEs | -0.22** | -0.16* | -0.03 | 0.29** | 0.41** |  |
| Physical EOEs | 0.03 | -0.05 | 0.12 | 0.34** |  |  |
| Social EOEs | -0.10 | -0.01 | -0.07 |  |  |  |
| Age | 0.01 | 0.01 |  |  |  |  |
| Training-  Duration | 0.25** |  |  |  |  |  |

Note: * p<.05; **p<.01

**Table 2**

*Association of EOEs with Team/Individual Sport and Group Training/Training Alone (ANOVAs)*

|  | Depended Variable | Df | *F* | Sig. |  | Cohen´s f |
| --- | --- | --- | --- | --- | --- | --- |
| Team/Individual Sport | Psychological EOEs | 1 | .53 | .47 |  | .05 |
|  | Negative EOEs | 1 | .67 | .41 |  | .05 |
|  | Physical EOEs | 1 | .00 | .95 |  | .00 |
|  | Overall EOEs | 1 | 1.34 | .25 |  | .08 |
| Group Training/ | Psychological EOEs | 1 | .32 | .57 |  | .04 |
| Training alone | Negative EOEs | 1 | .11 | .74 |  | .02 |
|  | Physical EOEs | 1 | 1.26 | .26 |  | .07 |
|  | Overall EOEs | 1 | 13.6 | .50 |  | .04 |

Note: Df = degree of freedom, *F* = test for statistical significance.

**Table 3**

*Descriptive Statistics on Subgroups*

|  | Psychological EOEs | | Negative EOEs | | Physical EOEs | | Social EOEs | |
| --- | --- | --- | --- | --- | --- | --- | --- | --- |
|  | *M* | *SD* | *M* | *SD* | *M* | *SD* | *M* | *SD* |
| Team  sport | 4.82 | 1.06 | 4.55 | 1.09 | 4.6 | 1.12 | 4 | 1.30 |
| Individual  sport | 4.72 | 1.09 | 4.44 | 1.05 | 4.59 | 1,25 | 3.42 | 1.29 |
| Training in a group | 4.80 | 1.06 | 4.52 | 1.10 | 4.55 | 1.15 | 3.93 | 1.30 |
| Training  alone | 4.71 | 1.12 | 4.46 | 1.00 | 4.75 | 1.23 | 3.21 | 1,26 |
| Female | 4.81 | 1.05 | 4.56 | 1.08 | 4.65 | 1.12 | 3.77 | 1.34 |
| Male | 4.74 | 1.12 | 4.42 | 1.06 | 4.52 | 1.26 | 3.73 | 1.30 |

Note: *M* = Mean value, *SD* = standard deviation.

**Table 4**

*Number of participants in the respective main sport*

| Main sport | Number of participants |
| --- | --- |
| Badminton | 1 |
| Basketball | 1 |
| Gym | 12 |
| Soccer | 6 |
| Handball | 11 |
| Martial Arts | 7 |
| Climbing | 6 |
| Running | 5 |
| Athletics | 6 |
| Cycling | 5 |
| Horse Riding | 43 |
| Swimming | 1 |
| Dancing | 1 |
| Tennis | 1 |
| Table Tennis | 15 |
| Gymnastics | 54 |
| Volleyball | 7 |
| Yoga | 10 |
| Skateboard | 3 |
| Ice Hockey | 8 |
| Field Hockey | 15 |
